# Promoting Neonatal Staff Nurses' Comfort and Involvement in End of Life and Bereavement Care

**DOI:** 10.1155/2013/365329

**Published:** 2013-03-27

**Authors:** Weihua Zhang, Betty S. Lane

**Affiliations:** ^1^Nell Hodgson Woodruff School of Nursing, Emory University, 1520 Clifton Road, Atlanta, GA 30322-4207, USA; ^2^Clayton State University, 2000 Clayton State Boulevard, Morrow, GA 30260-0285, USA

## Abstract

*Background*. Nurses who provide end of life and bereavement care to neonates and their families are potentially at risk for developing stress-related health problems. These health problems can negatively affect nurses' ability to care for their patients. *Purpose*. Nurses need to be knowledgeable about end of life and bereavement issues to provide quality care. This study sought to evaluate the effect of a bereavement seminar on the attitudes of nurses regarding end of life and palliative care of neonates. *Design*. A convenience sample of fourteen neonatal nurses completed a Bereavement/End of Life Attitudes about Care of Neonatal Nurses Scale after a bereavement seminar designed to provide information on end of life care. A pre- and posttest design with an intervention and control group was used to assess changes in nurse bereavement attitudes in relationship to comfort, role, and involvement. *Results*. After bereavement seminar, the seminar attendees had higher levels of comfort in providing end of life care than nurses in the control group (*t* = −0.214; *P* = 0.04). *Discussion*. Nurses' comfort levels can be improved by attending continuing education on end of life care and having their thoughts on ethical issues in end of life care acknowledged by their peers.

## 1. Introduction

Nurses who provide end of life and bereavement care to infants and their families are potentially at great risk for developing stress-related health problems. The emotional strain associated with end of life and bereavement care not only affects a nurse's health but can also affect relationships at home and with coworkers. The stress experienced by a nurse can even affect the quality of care provided to patients and parents [1, 2]. Moral distress is recognized as one of the major sources of stress for nurses who provide end of life care to infants. Factors that induce moral distress in nurses can result from providing care to infants who have withdrawal of treatment followed by death or extending futile treatment that induces unnecessary suffering [3]. There has been an increase in the proportion of deaths associated with decisions to forgo intensive care treatment from 23% in 1987 to 1988 to 64% in 1998 to 1999 [4]. More than half of neonatal deaths are associated with withdrawal of treatment [5], and treatment-related stressors to health care professionals have no doubt increased over the years as a result of advanced treatment options [6, 7].

Nurses need to be knowledgeable about bereavement and end of life issues and need to be comfortable in their interactions in order to provide quality bereavement and end of life care [8, 9]. It is a nurse's obligation to act on behalf of the patients and their families to ensure that the care provided is congruent with their preferences. A key to fulfilling this obligation is to cultivate relations with patients and their families [10]. The American Academy of Pediatrics committee on bioethics and its committee on hospital care stated in their integrated model of palliative care that palliative care should be offered at diagnosis and continued throughout the course of illness, whether care results in cure or death [11]. It is recommended that an uncertain prognosis should be a signal to initiate, rather than to delay, palliative care discussion [12]. Palliative care is not limited to end of life care; however, it ensures quality of end of life care. However, studies have indicated that neonatal nurses often are not comfortable with providing end of life care to infants. Some of the reasons associated with this discomfort are inadequate nursing education related to bereavement care in nursing school or in their place of employment [13, 14]. Other factors that may elevate the stress levels of nurses who provide end of life care to infants are provider's age, life experience, and clinical experiences that involve management of ethical dilemmas. It has been suggested that nurses' prior knowledge of end of life care is correlated with nurses' comfort in providing related care [15]. Nurses' moral distress can affect communication with parents, thus leading to changes in parents' perceptions of the quality of nursing care [16]. Nurses are viewed by families as being more supportive than physicians during grief. However, communication problems and a lack of awareness of cultural issues can serve as barrier that can cause parents to feel abandoned and that nurses lack sensitivity [17]. In addition, noneffective communication between health care professionals and infant's parents has been identified as one of the barriers that could cause unnecessary conflict in providing infant's care [18]. Issues related to the parents' cultural background can profoundly influence medical ethical decision making for infant's care [19, 20] and can result in moral distress in nurses. 

The literature indicates that nurses experience stress and moral discomfort in the provision of end of life and bereavement care. Ethical issues related to withdrawal of treatment and futile treatments are major contributors to nursing stress. Nurses' personal characteristics and experiences can impact their response to bereavement stress. Communication and awareness of cultural needs are also recognized as important in the care of patients and families. This study contributes to the literature because it is an intervention study which evaluates nurses' perceptions about their role, comfort, and involvement in end of life and bereavement care following an educational seminar. 

## 2. Purpose

In recognition of the stress experienced by nurses in providing end of life care to infants, the women's center of a level II hospital in the southeast offered a bereavement seminar to nurses who work in areas that experience infant deaths. The purpose of this study was to evaluate the effectiveness of the bereavement seminar on the attitudes of nurses regarding end of life care of neonates. The research questions were as follows: (1) what are the characteristics and attitudes of nurses who provide end of life/bereavement care to infants? (2) Is there a difference in nursing role, comfort, and involvement in providing end of life care between pre- and posttest scores of nurses who attended a bereavement seminar? (3) Will nurses who attend the educational seminar have higher scores in the domains of comfort, role, and involvement than the control group? 

## 3. Design and Methods

A pre- and posttest design with a nonequivalent control group and intervention group was used to compare the effect of the bereavement seminar on role, comfort, and involvement. A convenience sample of nurses was drawn from a community hospital in the southeastern United States. Nurses were eligible to participate in the study if they worked in Labor and Delivery, NICU, Mother Baby, Pediatrics, or the Emergency Department and worked directly with patients and were not in an administrative position. Nurses who attended the one-day education program were enrolled in this study as the intervention group. The control group comprised those who did not attend the education program, but worked in areas that provide end of life or bereavement care to infants. Nurses voluntarily signed up to attend the bereavement seminar and participate in the study. 

### 3.1. Instruments

Bereavement/End of life Attitudes About Care of Neonatal Nurses Scale (BEACONNS) was the instrument used for this study [21]. Permission to use the BEACONNS Scale was obtained from Engler and Associates, the researchers who had developed the instrument. Reliability for the instrument when used in a descriptive study ranged between 0.81 and 0.95 [21]; it has not yet been used in quasiexperimental design. There are three domains in the BEACONNS scale: (1) the comfort level of handling end of life and bereavement care, (2) role of propensity in providing end of life and bereavement care, (3) tendency of allowing family involvement in providing end of life care.

#### 3.1.1. Comfort Scale

The comfort scale measures nurses' perceptions about the degree of comfort they felt with various aspects of bereavement/end of life care. There are 19 Likert-scale items in this subscale, with scores ranging from 1 (very uncomfortable) to 5 (very comfortable). The 19 items are summed for a total score. Higher total scores indicate greater comfort levels with providing end of life care. The reliability for the comfort scale Cronbach *α* is 0.95 [21]. 

#### 3.1.2. Role Scale

 The 18-item Likert-type role scale measures nurses' perceptions of their roles with families of critically ill and/or dying infants; scores range from 1 (strongly disagree) to 5 (strongly agree). Some items required reverse scoring. The 18 items are summed for a total score. Higher total scores indicate more supportive roles in facilitating families' involvement in end of life/bereavement care. The reliability of the role scale Cronbach *α* is 0.85 [21].

#### 3.1.3. Involvement Scale

The 14-item involvement scale measures nurses' ratings of the importance of various factors relative to their involvement with patients and families. The Likert-type scale ranges from 1 (very unimportant) to 5 (very important), and the items are summed for a total score. The higher the total score, the more the involvement nurses thought they should have with their patients and families. The reliability for the involvement scale Cronbach *α* is 0.85 [21]. 

#### 3.1.4. Other Demographic Variables

In the BEACONNS instrument, there are additional items that acquire information about a nurse's educational background, ethnicity, prior experience with infant death, and significant personal loss. 

### 3.2. Procedure

Institutional approval for conducting the study was obtained prior to the study from the Professional Development Council from the institution where all nurses were recruited. Information obtained was confidential and findings were reported in group format. An eight-hour end of life/bereavement seminar was developed by a planning committee of staff representatives from the areas that experience fetal and infant deaths. The seminar was entitled “How do I make IT feel better?” “IT” refers to the physiological and psychological impact of loss on both family and staff. The objective of the seminar was to alleviate this impact and reduce staff's moral distress. The seminar focused on developing strategies to support the caregiver and family in dealing with their grief and to utilize support services in the community and hospital. It also focused on dealing with ethics issues and improving communication skills. Flyers and brochures were sent out to the Labor and Delivery, Mother-Baby, NICU, Pediatrics, and the Emergency Departments advertising the workshop. 

 Nurses who were direct patient care providers were invited to participate in the study through distribution of a flyer and communication at staff meetings. The morning of the bereavement seminar prior to the start of the program the nurses who wished to participate in the study signed a consent form and completed the BEACONNS survey. This group represented the intervention group and the survey completed before the seminar was the pretest survey. The BEACONNS survey and a consent form were also distributed to these selected departments and all staff nurses were invited to complete the survey as the control group. Two months after the bereavement program, the intervention group completed a second BEACONNS scale as the posttest survey. 

## 4. Results

### 4.1. Characteristics of Sample

Data were analyzed using SPSS version 14. Descriptive statistics were performed to measure frequencies, means, and percentages. Independent and paired *t*-tests were conducted to test for statistical mean differences between the pre- and the posttest group. An independent *t*-test was used to compare the differences between the intervention and the control groups. A total of 63 nurses participated in the study. The majority was married (83%), Protestant (34.4%), and Caucasian (82.3%) and had children (84%). Most held a BSN (46.8%) or an ADN (41.9%) degree and worked in the NICU (38.7%). The average age of the nurses who participated in the study was 39.42 years (SD 10.5). The average years of working experience as a nurse were 5.29 years (SD 8.2). Nineteen percent had experienced a significant personal loss in the past year. Seventy-one percent had end of life/bereavement care in their basic nursing education. Less than half (43.5%) were satisfied or very satisfied with their prior end of life nursing education. Slightly more than a fourth (28.6%) had attended continuing education courses on end of life/bereavement care. Over three-fourths had cared for dying infants. When asked who was most important in providing support to families of critically ill infants on a daily basis participants indicated that nurses (mean = 4.70) were most important (5 indicates the most important role, and 1 indicates the least important role), followed by physicians (mean = 4.43) and chaplains (mean = 4.05). 

### 4.2. Data Analysis

To answer the research question “was there a difference between pre- and posttest scores of nurses who attended the bereavement seminar?”, a paired *t*-test was used. Data from nurses prior to the seminar and two months after the seminar were compared. Nineteen participants completed the pretest questionnaire; only 14 participants returned the posttest questionnaire of nurses' comfort level with end of life/bereavement care being significantly increased (*t* = −3.37, *P* = 0.01). However, postrole scores were not significantly different from the prerole scores (*t* = 1.09, *P* = 0.30). Likewise, postinvolvement scores were not significantly different from the preinvolvement scores (*t* = −0.19, = 0.84). Boxplots and paired “ladder” plots were used to provide illustrative support for these findings.

An independent *t*-test was performed in order to answer the third research question, “is there a difference in the three domains between the nurse groups who attended the workshop and the group that had not attended the workshop?” The first survey prior to the seminar was used to analyze the difference. No differences were found between these two groups in comfort level (*t* = 0.77, *P* = 0.44), role (*t* = 0.09, *P* = 0.92), and involvement (*t* = 0.24, *P* = 0.82). Further, no difference was detected in those who signed up for the class and those who did not sign up for the class in whether they had previous training in end of life care/bereavement care. However, the difference in satisfaction with previous end of life/bereavement care training was significant in those who came to the workshop and those who did not come to the workshop. Those who came to the workshop were less satisfied with their previous end of life/bereavement care training than those who did not come (*t* = −2.21, *P* = 0.03). 

## 5. Discussion and Conclusions

The majority of the sample was Caucasian, Christian, married, and had children. Slightly more of the staff was BSN prepared and the mean age was 39.42. These demographic characteristics were similar to the participants in a study by Engler et al. [21] that looked at neonatal staff and advanced practice nurses' perceptions of bereavement/end of life care. However, the nurses in Engler's study had worked on average over twice as long (16.4 versus 5.29 years) and had a greater percentage of nurses who had experienced a significant loss in the past year (31 versus 19 percent). The difference in the number of years worked may reflect the transient nature of the southeast population and the location of several nursing programs near the hospital. The greater percentage of nurses who had experienced loss in the Engler study may be due to that sample being slightly older than the nurses in the current study. 

 Most nurses had received end of life care in their basic nursing programs; interestingly, less than half were pleased with these offerings. Those who came to the workshop were less satisfied with their previous end of life nursing education training, which may have been a motivator for their attendance at the bereavement workshop. These nurses in the study have been working in areas that experience neonatal deaths; surprisingly only a third had attended continuing education on end of life/bereavement care. 

Another important finding was that the intervention group's comfort level had increased at the posttest which was 2 months after the seminar. This supports the findings of Fredrickson et al. who found that a nurse's prior education about end of life care increases comfort and decreases moral distress. The comfort level increments could be the result of the seminar content as well as the interactions among nurses during the daylong seminar. A noticeable observation from this seminar is that nurses are gaining new knowledge while they were sharing their own experience and acknowledging their peers. There was not a significant difference in role perceptions between or among groups. The participants felt that their role in providing support to families of critically ill infants on a daily basis was more important than that provided by physicians. Involvement was not significantly different between the two groups. A lack of statistical difference between the control and intervention group may have been due to the small sample size. Also, nurses who did not attend the seminar may have already felt at ease in the areas of comfort, role, and involvement. In addition, the reliability of the involvement scale may have been affected by the phrasing of the directional stem on the Likert scale; many nurses found the involvement scale confusing. 

The results of this study supported the use of a bereavement seminar to increase nurse comfort levels in the provision of end of life/bereavement care to infants and families in the intervention group. Nurses overall were not satisfied with the end of life/bereavement education they had received in their undergraduate nursing programs. This suggests a need for nursing schools to ensure their curricula should include content on caring for critically ill and dying neonates and their families. Nurses who work on units with neonates need supportive continuing education, since three-fourths of the nurses had experienced neonatal deaths, yet only one-fourth had attended continuing education on end of life/bereavement care. Future intervention studies are needed with larger nurse populations in order to evaluate the effectiveness of end of life/bereavement education on comfort and involvement in end of life care. Studies should also assess whether greater nurse comfort scores impact family satisfaction with end of life/bereavement of their neonate.
